# Complete Genome Sequences of Grapevine Yellow Speckle Viroid 1 and Hop Stunt Viroid Assembled from the Transcriptome of *Ixeridium dentatum* Plants

**DOI:** 10.1128/genomeA.01248-15

**Published:** 2015-10-29

**Authors:** Joong-Hwan Lee, Seungmo Lim, Seung-Won Lee, Ran Hee Yoo, Davaajargal Igori, Fumei Zhao, Youngnam Yoon, Su-Heon Lee, Jae Sun Moon

**Affiliations:** a Institute for Bioresources Research, Gyeongsangbuk-do Agricultural Research & Extension Services, Andong, Republic of Korea; b Plant Systems Engineering Research Center, Korea Research Institute of Bioscience and Biotechnology, Daejeon, Republic of Korea; c Biosystems and Bioengineering Program, University of Science and Technology, Daejeon, Republic of Korea; d Bioinformatics Team & Research Institute, SeqGenesis, Daejeon, Republic of Korea; e School of Applied Biosciences, Kyungpook National University, Daegu, Republic of Korea; f Crop Production Technology Research Division, NICS, RDA, Miryang, Republic of Korea; g Institute of Plant Medicine, Kyungpook National University, Daegu, Republic of Korea

## Abstract

Here, we report complete genome sequences of grapevine yellow speckle viroid 1 (GYSVd1) and hop stunt viroid (HSVd), members of the family *Pospiviroidae*, assembled from the transcriptome data generated from *Ixeridium dentatum* plants. To our knowledge, this is the first report of GYSVd1 and HSVd in *I. dentatum*.

## GENOME ANNOUNCEMENT

Viroids have a small (about 250 to 400 nucleotides [nt]), single-stranded, and circular RNA genome that does not code for any proteins (1, 2). So far, approximately 30 different species of viroids belonging to two families, *Pospiviroidae* and *Avsunviroidae*, have been identified since potato spindle tuber viroid was discovered a few decades ago (1). Viroids have distinguishing features, such as secondary structure, replicate site, rolling circle mechanism, ribozyme activity, and host range, which determine the criteria for viroid classification (2).

In June 2013, *Ixeridium dentatum* plants were randomly collected in Andong, South Korea. Because diseases caused by a virus or viroid in *I. dentatum*, which can be used as food and medicine, have not been well studied, transcriptome data generated from *I. dentatum* plants using a next-generation sequencing (NGS) method were analyzed. To construct a library with the TruSeq RNA sample prep kit (Illumina, San Diego, CA, USA), total RNA was extracted from one pool of all *I. dentatum* samples using TRI reagent (Molecular Research Center, Cincinnati, OH, USA), and rRNA was removed from the total RNA using Ribo-Zero rRNA removal kits (plant leaf) (Epicentre, Madison, WI, USA). The size and purity of the constructed library were measured by BluePippin 2% agarose gel cassettes (Sage Science, Beverly, MA) and the Agilent 2100 Bioanalyzer (Agilent Technologies, Santa Clara, CA, USA), respectively. Raw data were produced from the Illumina HiSeq 2500 paired-end RNA sequencing by the Theragen Etex Bio Institute (Suwon, South Korea). The raw data processing, including assembly of reads into contigs and annotation of contigs using the NCBI database for sequence similarity searches, was conducted by SeqGenesis (Daejeon, South Korea).

Interestingly, two contigs were highly matched to grapevine yellow speckle viroid 1 (GYSVd1) (genus *Apscaviroid*) and hop stunt viroid (HSVd) (genus *Hostuviroid*). They covered the whole genome sequence of each viroid. The complete genome sequence of GYSVd1, with a length of 366 nt, named GYSVd1 isolate AD (GenBank accession no. KT725430), shared 100% identity (100% query coverage) with GYSVd1 isolated from grapevine in Thailand (accession no. KP010005), and that of HSVd, with a length of 297 nt, named HSVd isolate AD (GenBank accession no. KT725429), shared 100% identity (100% query coverage) with HSVd isolated from a grapevine in Chile (accession no. KF007325). The two viroids are widely known to infect grapevines (*Vitis vinifera*), and diseases caused by them might lower their commercial value (3–5). In many cases, grapevines are infected with multiple viroids (6).

In this work, we identified two members of the family *Pospiviroidae*, GYSVd1 and HSVd, using the transcriptome analysis of *I. dentatum* plants. To our knowledge, this is the first report of the presence of GYSVd1 and HSVd in *I. dentatum*.

### Nucleotide sequence accession numbers. 

The complete genome sequences of grapevine yellow speckle viroid 1 isolate AD and hop stunt viroid isolate AD have been deposited in GenBank under the accession numbers KT725430 and KT725429, respectively.
